# Maternal and Neonatal Outcomes in Pregnancy Complicated with Pulmonary Hypertension

**DOI:** 10.31083/j.rcm2305172

**Published:** 2022-05-12

**Authors:** Hua Liao, Qing Hu, Chunyan Deng, Xiaodong Wang, Haiyan Yu

**Affiliations:** ^1^Department of Obstetrics and Gynecology, West China Second University Hospital, Sichuan University, 610041 Chengdu, Sichuan, China; ^2^Key Laboratory of Birth Defects and Related Diseases of Women and Children (Sichuan University), Ministry of Education, 610041 Chengdu, Sichuan, China

**Keywords:** pulmonary hypertension, heart disease, pregnancy complication, maternal and neonatal outcomes

## Abstract

**Background:**

Pulmonary hypertension (PH) is a life-threatening disease with significant maternal morbidity and mortality.

**Methods:**

To assess pregnancy and neonatal outcomes and determine the risk factors for adverse maternal and neonatal outcomes in women with pulmonary hypertension (PH), a retrospective analysis was carried out examining 71 pregnancies in patients with PH who delivered at a tertiary care center in West China between January 2011 and May 2016.

**Results:**

One pregnancy resulted in spontaneous abortion and six resulted in terminated abortions. Cardiac complications were encountered in 16.9% including three maternal mortalities. At least one pregnancy complication occurred in 28.2% of all the pregnancies. Diagnosis after the third trimester, severe PH and/or right ventricular systolic dysfunction were predictive of adverse fetal/neonatal events. A history of prior cardiac events and right ventricular systolic dysfunction and/or baseline New York Heart Association (NYHA) class III or IV were the main predictive factors of adverse maternal cardiac events.

**Conclusions:**

In our study, we found that PH poses high risks for maternal and fetal morbidity and mortality. A detailed pre-pregnancy baseline assessment is strongly recommended in women with PH to identify those with the highest risk and subsequently guide clinical management.

## 1. Introduction

Pulmonary hypertension (PH) is defined as an elevation in mean pulmonary arterial pressure to ≥25 mmHg at rest as assessed by right heart catheterization [1]. The 6th World Symposium on Pulmonary Hypertension recommended the upper limit of normal mean pulmonary arterial pressure to be 20 mmHg [2]. The worldwide prevalence of PH is estimated to be 1% overall, rising to 10% in elderly people over 65 years old, and to at least 50% in patients with heart failure [3]. Based on etiology, PH can be categorized into 5 groups. Group 1 is pulmonary arterial hypertension (PAH), the causes of which include idiopathic, heritable, drug- and toxin-induced, connective tissue diseases, human immunodeficiency virus (HIV) infection, portal hypertension, congenital heart disease. Group 2 refers to pulmonary hypertension associated with left-sided heart diseases, which is usually a consequence of heart failure or valvular heart disease. Pulmonary hypertension due to lung disease, pulmonary obstructive disease such as pulmonary embolism, and unclear mechanisms, are categorized into Groups 3, 4, and 5 respectively [4]. The overall estimated prevalence of PAH is ranging from 10 to 52 cases per million worldwide [5]. PAH is a life-threatening disease with increased pulmonary vascular resistance leading to right ventricular failure and even death, with a survival rate of 68% to 93% at 1 year and 39% to 77% at 3 years [3, 6].

In women of childbearing age, the most common cause of PH relates to the late consequences of Eisenmenger’s syndrome with heart disease. Hemodynamic changes during pregnancy and the peripartum, poor adaptation of the right heart, and insufficient compliance of pulmonary vasculature seem to be associated with poor tolerance in these patients. The maternal mortality rates of patients with PH have been reported to be as high as 30–56% in the last decade [7]. Therefore, pregnancy in the presence of PAH is contraindicated and recommended for early termination within current guidelines [2]. However, for patients with a high desire for pregnancy and for those who first presented with PH after conception, rational treatment is important to improve clinical outcomes.

The management of PH has evolved considerably in recent years. Due to the utilization of multidisciplinary therapies, maternal mortality rates in PH have been documented as declining to 9–25% [8, 9].

Successful pregnancies have been reported in small series or case reports to date, but data are incomplete for maternal and fetal outcomes in these patients, especially in developing countries. Therefore, the purpose of this study is to determine maternal and fetal outcomes in patients with pulmonary hypertension in west China.

## 2. Materials and Methods

This study was approved by the Institutional Review Board of West China Second University Hospital. The medical records of pregnant women with Hospitalized at West China Second University Hospital from January 2011 and May 2016 were retrospectively analyzed.

### 2.1 Subjects

West China Second University Hospital is the largest regional tertiary referral center in West China, handling more than 12,000 deliveries per year. The inclusion criteria were as follows: (1) patients with complete medical records; (2) patients with at least 1 echocardiographic investigation and with a recorded peak tricuspid regurgitation velocity (TRV) to derive systolic pulmonary artery pressure (PASP); (3) patients with PASP ≥30 mmHg on average. A total of 71 pregnant women with PH were identified, most of whom were referred from other local hospitals. Written permissions were obtained from patients or relatives for the use of medical records, images and patient information.

The antenatal records and medical records for the mothers and newborns were investigated retrospectively. All women were followed up with until 6 weeks postpartum. The medical records were reviewed by two authors (HL, QH) independently. All discrepancies were resolved by rechecking the source papers and discussion between the two authors, and, if necessary, consultation by all authors, with full consensus prior to inclusion.

### 2.2 Management of PH

The patient’s condition was continuously assessed by the multidisciplinary teams, consisting of obstetricians, cardiologists, cardiac ultrasound specialists, midwifes, anesthetists, intensive care physicians and neonatologist. A diagnosis of PH was established by clinical history, physical examination, and echocardiography. Transthoracic echocardiography (TTE) was used to assess pulmonary arterial systolic pressure and to identify the diagnosis of pulmonary hypertension (PH).

Transthoracic echocardiography was performed for all the patients. The estimation of pulmonary arterial pressure was based on the Doppler pressure gradients across the tricuspid valve and a modification of the Bernoulli principle [2]. Patients were classified according to baseline resting systolic pulmonary artery pressure as mild PH (PASP 30– 49 mmHg), moderate PH (PASP 50–79 mmHg), or severe PH (PASP ≥80 mmHg) [10].

### 2.3 Data Collection

The following data were recorded for each patient: maternal age, parity, ethnicity, residence, etiology of pulmonary hypertension, prior cardiac interventions, prior cardiac events (arrhythmias, transient ischemic attack, heart failure or stroke), New York Heart Association (NYHA) functional class, change in the cardiac status during pregnancy, new onset or aggravation of cardiac complications, cardiac and PH medications, obstetric complications, duration of hospitalization, gestational age at delivery, delivery mode, and anesthetic management. birth weight, Apgar scores, sex, fetal and neonatal complications, attendance to the neonatal intensive care unit (NICU), and perinatal mortality were also investigated in all cases.

### 2.4 Statistical Analyses

Categorical variables were expressed as the number (%) per group. Continuous variables of normal distribution were described as mean ± standard deviation (SD) or median (25th quartile, 75th quartile) of nonnormal distribution. Normally distributed continuous data were compared using unpaired Student’s *t*-test, whereas nonnormally distributed continuous data were compared using Mann–Whitney U-test. Data for categorical variables were analyzed using the chi-square test or Fisher’s exact test, with significance set at *p *< 0.05. Univariate analysis was initially performed to determine variables with a significant association with adverse maternal or neonatal events. Multivariable analysis was performed stepwise, leaving only the significantly associated confounders to the final models. Confidence intervals (CI) were evaluated at 95%. All statistical analysis was performed using the SPSS 22.0 software (SPSS for Windows, version 22.0; SPSS, Inc., Chicago, IL, USA).

## 3. Results

### 3.1 Clinical Characteristics and Underlying Diseases in Patients with PH

The 71 pregnant patients with PH were all singleton pregnancies. The mean maternal age was 27 years (range: 17–38 years). PH was diagnosed before pregnancy in 36.6% of cases, 22.6% before 28 gestational weeks during pregnancy, and 40.8% after 28 gestational weeks. 15 cases had mild PH, 28 cases had moderate PH, and 28 cases had severe PH. The NYHA functional classifications at the first visit were 90.1% for NYHA stages I–II and 9.9% for NYHA stages III–IV. According to the NYHA functional classification at the time of admission, 28.2%, 54.9%, and 16.9% of the patients were in NYHA stages I–II, III, and IV, respectively. The detailed maternal characteristics at the time of admission of all patients are shown in Table 1. There was no significant difference among the 3 groups regarding gravida, poor antenatal care, or NYHA functional classification.

**Table 1.** **Maternal characteristics of patients with PH**.

Variable		Total (n = 71)	Mild PH (n = 15)	Moderate PH (n = 28)	Severe PH (n = 28)	*p *value
PASP (mmHg) #		70 (52, 92)	43 (41, 45)	70 (63, 76)	102 (100, 118)	<0.001
Maternal Age (years)*		27.0 ± 6.8	20.1 ± 5.2	25.2 ± 4.9	32.7 ± 5.3	0.035
Residence						
	Urban, n (%)	46 (64.8)	13 (86.7)	7 (25.0)	26 (92.9)	
	Rural, n (%)	25 (35.2)	2 (13.3)	21 (75.0)	2 (7.1)	0.000
Primipara, n (%)		29 (40.8)	5 (33.3)	11 (39.3)	13 (46.4)	0.674
Poor antenatal care attenders**, n (%)		34 (47.9)	6 (40.0)	16 (57.1)	12 (42.9)	0.51
Previous cardiovascular ${\mathrm{events}}^{\text{a}}$, n (%)		8 (11.3)	1 (6.7)	3 (10.7)	4 (14.3)	0.894
Previous obstetric ${\mathrm{events}}^{\text{b}}$, n (%)		23 (32.4)	6 (40.0)	8 (28.6)	9 (32.1)	0.76
Time to diagnose, n (%)						
Before pregnancy, n (%)		26 (36.6)	9 (60.0)	6 (21.4)	11 (39.3)	0.04
During pregnancy, n (%)						
	First trimester, n (%)	4 (5.6)	0 (0.0)	1 (3.6)	3 (10.7)	0.526
	Second trimester, n (%)	12 (16.9)	1 (6.7)	6 (21.4)	5 (17.9)	0.519
	Third trimester, n (%)	29 (40.8)	5 (33.3)	15 (53.6)	9 (32.1)	0.238
NYHA functional classification						
	Stage I–II, n (%)	20 (28.2)	7 (46.7)	8 (28.6)	5 (17.9)	0.150
	Stage III, n (%)	39 (54.9)	6 (40.0)	14 (50.0)	19 (67.9)	0.198
	Stage IV, n (%)	12 (16.9)	2 (13.3)	6 (21.4)	4 (14.3)	0.778

Data presented are # median (25th quartile, 75th quartile); * mean value ± SD. **Poor antenatal care attenders were defined as women having first antenatal appointments at 18 weeks gestation or later; or missing 2 appointments without notification; or without preconception counseling. (a) Previous Cardiovascular events refer to a history of previous cardiovascular events, including pulmonary edema, cardiac arrest, stroke, and symptomatic persistent arrhythmias requiring therapy. (b) Previous obstetric events refer to a history of previous pregnancy complications including pre-eclampsia, postpartum hemorrhage, spontaneous preterm birth and intrauterine growth restriction. PH, pulmonary hypertension; PASP, systolic pulmonary artery pressure.

The underlying diseases of PH in the study population were congenital heart disease (CHD, 70.4%), rheumatic heart disease (RHD, 22.5%), idiopathic pulmonary arterial hypertension (2.8%), thalassemia (2.8%), and acute peripartum cardiomyopathy (1.4%). Detailed information on these cases is shown in Table 2.

**Table 2.** **Underlying diseases in patients with PH**.

		No. (%)
Rheumatic heart disease		16 (22.5)
	Predominate mitral stenosis	5 (7.0)
	Predominate mitral regurgitation	1 (1.4)
	Mixed mitral diseases	2 (2.8)
	Multivalvular lesions	6 (8.5)
	Prosthetic valves	2 (2.8)
Congenital heart disease		50 (70.4)
	Atrial septal defect	20 (28.2)
	Ventricular septal defect	12 (16.9)
	ASD combined with VSD	4 (5.6)
	Patent ductus arteriosus	$4^{\text{b}}$ (5.6)
	Bicuspid aorta	1 (1.4)
	Pulmonary stenosis	1 (1.4)
	Anomalous pulmonary venous drainage	3 (4.2)
	Transposition of the great arteries	1 (1.4)
	Endocardial Cushion Defect	1 (1.4)
	Tetralogy of Fallot	$3^{\text{c}}$ (4.2)
${\mathrm{Others}}^{\text{a}}$		5 (7.0)

ASD, Atrial septal defect; VSD, ventricular septal defect. (a) Including idiopathic pulmonary hypertension (2), thalassemia (2) and acute peripartum cardiomyopathy (1). (b) One patient received operation before pregnancy. (c) Two patients received operation before pregnancy.

### 3.2 Maternal Cardiac Outcomes

A cardiac event complicated 12 patients (16.9%), with 3 cardiac maternal deaths (4.23%) (Table 3). Most cardiac events (92%) occurred during the antepartum period. The most common cardiac complication was arrhythmia, documented in 9 pregnancies.

**Table 3.** **Maternal adverse cardiac outcomes during ${\mathrm{pregnancy}}^{\text{𝐚}}$**.

	No	Mild PH	Moderate PH	Severe PH	*p* value
		(n = 15), n (%)	(n = 28), n (%)	(n = 28), n (%)	
Cardiac Death	3	1 (6.67)	1 (3.57)	1 (3.57)	0.99
Heart failure	6	1(6.67)	3 (10.7)	2 (7.14)	0.99
Arrhythmia	9	1(6.67)	5 (17.9)	3 (10.7)	0.661
Stroke/${\mathrm{TIA}}^{\text{b}}$	1	0 (0.0)	1 (3.57)	0 (0.0)	0.99
Total events	19	3 (20.0)	10 (35.7)	6 (21.4)	0.484

(a) Events are not mutually exclusive. 12 pregnancies complicated by 1 or more events. (b) TIA: transient ischemic attack. PH, pulmonary hypertension.

Maternal mortality was observed in three cases (4.23%). Two of these occurred after C-section in the early postpartum period due to recurrent sustained cardiac arrhythmia and heart failure. Another occurred at 35 gestational weeks due to persistent atrial fibrillation and progressive heart failure.

Risk factors associated with maternal adverse cardiac event during pregnancy in univariate analysis are listed in Table 4. After multivariable analysis, independent predictors of adverse cardiac event during pregnancy included prior cardiac events and right ventricular systolic dysfunction and/or baseline NYHA function class III or IV.

**Table 4.** **Predictors of maternal adverse cardiac events in pregnancy women with PH (n = 71)**.

		Adverse cardiac events	No adverse cardiac events	*p* value
		(n = 12)	(n = 59)	
Univariate analysis				
	Multiparity, n (%)	9 (75.0)	33 (55.9)	0.34
	35 years or older, n (%)	2 (16.7)	8 (13.6)	0.67
	Poor antenatal care, n (%)	9 (75.0)	25 (42.4)	0.06
	Prior adverse cardiac events, n (%)	6 (50.0)	2 (3.4)	0.001
	Baseline NYHA function class III or IV, n (%)	5 (41.7)	2 (3.4)	0.001
	Moderate or severe PH, n (%)	10 (83.3)	46 (78.0)	0.98
	Maternal oxygen saturation <90%, n (%)	4 (33.3)	1 (1.7)	0.002
	Right ventricular systolic dysfunction, n (%)	8 (66.7)	6 (10.2)	<0.001
Multivariate analysis				
	Prior adverse cardiac events			0.031
	Right ventricular systolic dysfunction and/or baseline NYHA function class III or IV			0.003

PH, pulmonary hypertension; NYHA, New York Heart Association.

### 3.3 Obstetric and Fetal/Neonatal Outcomes

One pregnancy (1.4%) resulted in spontaneous abortion and six (8.5%) were electively terminated. At least one adverse obstetric event occurred in 28.2% of the 71 pregnancies, the most common obstetric events including placenta abruptio (n = 4), placenta previa (n = 3), and postpartum hemorrhage (n = 3). Higher rate of preeclampsia was found among women with mild PH than those with moderate or severe PH. No significant differences were noted in terms of other obstetric and perinatal outcomes, such as placenta abruption, placenta previa, postpartum hemorrhage, non-cardiac maternal death, preterm birth, small for gestational age, fetal distress, fetal demise, and neonatal death (Table 5).

**Table 5.** **Obstetric and fetal/neonatal outcomes**.

		Total	Mild PH	Moderate or severe PH	*p* value
		(n = 71)	(n = 15)	(n = 56)	
Aborted pregnancy, n (%)		7 (9.9)	0 (0.0)	7 (12.5)	0.332
	Spontaneous, n (%)	1 (1.4)	0 (0.0)	1 (1.8)	0.99
	Induced, n (%)	6 (8.5)	0 (0.0)	6 (10.7)	0.185
Obstetric events (71)					
	Preeclampsia, n (%)	2 (2.8)	2 (13.3)	0 (0.0)	0.006
	Placenta abruption, n (%)	4 (5.6)	0 (0.0)	4 (7.1)	0.572
	Placenta previa, n (%)	3 (4.2)	1 (6.7)	2 (3.6)	0.507
	Postpartum haemorrhage, n (%)	3 (4.2)	1 (6.7)	2 (3.6)	0.507
	Noncardiac maternal death, n (%)	0 (0.0)	0 (0.0)	0 (0.0)	
Fetal/neonatal events* (64)					
	Preterm birth, n (%)	22 (34.4)	4 (26.7)	18 (36.7)	0.549
	Small for gestational age, n (%)	12 (18.8)	1 (6.7)	11 (22.4)	0.265
	Fetal distress, n (%)	6 (9.4)	1 (6.7)	5 (10.2)	0.99
	Neonatal asphyxia, n (%)	12 (18.7)	2 (13.3)	10 (20.4)	0.424
	Respiratory distress syndrome, n (%)	7 (10.9)	2 (13.3)	5 (10.2)	0.662
	Intrauterine fetal demise, n (%)	7 (10.9)	2 (13.3)	5 (10.2)	0.662
	Neonatal death, n (%)	2 (3.1)	0 (0.0)	2 (4.1)	0.999

* Fetal/neonatal outcomes in the 64 completed pregnancies. PH, pulmonary hypertension.

Using a multivariate analysis with backward elimination, the following factors were significantly associated with adverse fetal/neonatal outcomes during pregnancy: diagnosis of PH after the third trimester; severe PH and/or right ventricular systolic dysfunction (Table 6).

**Table 6.** **Maternal predictors of adverse neonatal events (n = 64)**.

		Adverse neonatal events	No adverse neonatal events	*p *value
		(n = 41)	(n = 23)	
Univariate analysis				
	Poor antenatal care, n (%)	26 (63.4)	8 (34.8)	0.038
	Diagnosis after the third trimester, n (%)	23 (56.1)	6 (26.1)	0.035
	Baseline NYHA class III or IV, n (%)	6 (14.6)	1 (4.3)	0.406
	Severe PH, n (%)	20 (48.8)	3 (13.0)	0.016
	Right ventricular systolic dysfunction, n (%)	13 (31.7)	1 (8.7)	0.022
	Symptomatic arrhythmia, n (%)	8 (19.5)	1 (4.3)	0.14
Multivariate analysis				
	Diagnosis after the third trimester			0.019
	Severe PH and/or right ventricular systolic dysfunction			0.001

NYHA, New York Heart Association; PH, pulmonary hypertension.

### 3.4 Management of Successful Pregnancies

Before pregnancy, women with a history of heart disease were assessed by cardiovascular and obstetric specialists to evaluate the maternal and fetal risks and make individual treatment plans. High-risk patients not suitable for pregnancy were recommended for therapeutic abortions. There were six therapeutic abortions in our study population.

Women selected to continue pregnancy were managed by a multidisciplinary medical team. Frequent prenatal obstetric and cardiac visits (typically at 2–4 weeks intervals before 28 weeks and weekly thereafter) were recommended to allow for close monitoring of patients. Ultrasounds and echocardiography were performed every 2–4 weeks before 24 weeks and weekly thereafter. The patients’ mean gestational age at admission was 33.8 ± 4.5 and mean gestational age at delivery was 36.2 ± 3.1 weeks. In our series, major vascular drugs such as calcium channel blockers, prostacyclins, and PDE-5 inhibitors were applied to reduce pulmonary vascular resistance. Two women had a spontaneous preterm vaginal delivery and the other 62 patients had elective cesarean section procedures under epidural, general, or combined spinal-epidural anesthesia. Details of the management for women with PH are provided in Table 7.

**Table 7.** Management of pregnant women with PH (n = 64) *.

		Total	Mild PH	Moderate PH	Severe PH
		(n = 64)	(n = 15)	(n = 26)	(n = 23)
Hospital admission (weeks)		33.8 ± 4.5	35.2 ± 4.6	34.5 ± 2.3	31.8 ± 5.2
Delivery (weeks)		36.2 ± 3.1	37.3 ± 3.6	36.4 ± 2.9	34.8 ± 4.7
	Vaginal delivery, n (%)	2 (3.1)	0 (0.0)	0 (0.0)	2 (8.7)
	Surgical delivery, n (%)	62 (96.9)	15 (100.0)	26 (100.0)	21 (91.3)
Anesthesia					
	General anesthesia, n (%)	24 (37.5)	5 (33.3)	9 (34.6)	10 (43.5)
	Epidural anesthesia, n (%)	27 (42.2)	7 (46.7)	12 (46.2)	8 (34.8)
	CSEA, n (%)	11 (17.2)	3 (20.0)	5 (19.2)	3 (13.0)
Antithrombotic therapy, n (%)		11 (17.2)	2 (13.3)	4 (15.4)	5 (21.7)
ICU admission, n (%)		16 (25.0)	3 (20.0)	5 (19.2)	8 (34.8)

* Not included 7 abortions (6 therapeutic induced abortions and 1 spontaneous abortion). PH, pulmonary hypertension; CSEA, combined spinal-epidural anesthesia; ICU, intensive care unit.

## 4. Discussion

Our current study demonstrated that a history of prior cardiac events and right ventricular systolic dysfunction and/or baseline NYHA function class III or IV were the main determinants of adverse maternal cardiac events in pregnancies complicated with PH. Furthermore, late diagnosis and maternal severe PH and/or right ventricular systolic dysfunction were significantly associated with adverse fetal/neonatal outcomes in our series. Some studies have identified risk factors for adverse maternal outcomes in pregnancies complicated by heart diseases, including baseline NYHA functional class III or IV, and prior cardiac events [11]. Our findings in pregnancies complicated with PH were similar.

Right heart catheterization (RHC) is known as the gold standard for the diagnosis of PH [12]. The essential role of RHC in the diagnosis, management decision making, and assessment of the response to interventional procedures in patients with PH is well established. However, as an invasive technique, RHC also has catheter-related complications. In our study, transthoracic echocardiography (TTE) is used to diagnose PH. TTE is noninvasive, reproducible, and is the preferred imaging method during pregnancy. TTE has a pooled sensitivity of 85%, and a pooled specificity of 74% in the diagnosis of PH [13].

Pregnancy places a significantly physiologic load on the cardiovascular system with circulating blood volume increased by nearly 50%. The increased intravascular volume in those whose cardiac output is limited predisposes them to acute pulmonary edema, congestive cardiac failure, and a high mortality rate. The maternal mortality rate was 4.23% in our study, which is consistent with a previous study that reported an incidence of 4.8–9% [8, 14]. This was not consistent with the previous studies which revealed significantly higher maternal mortality rates of around 30% for Eisenmenger’s and primary PH, and 52% for secondary PH [8]. This might be due to the comparatively small number of idiopathic PH (2.8%) and Eisenmenger’s syndrome cases (4.2%) in our study population.

The neonatal outcomes were poor in our series, with a high incidence of preterm birth at 34.4%, and cases of small for gestational age (SGA) at 18.8%. Increased risk of SGA was found to be especially high for women with moderate or severe PH (with PASP higher than 50 mmHg), as opposed to those with mild PH (with PASP less than 49 mmHg). In a prior series of 20 women with PH, two patients in the pulmonary hypertension group had congestive cardiac failure, which was not significantly higher than those without pulmonary hypertension, but one was associated with the only maternal death and the other with the only perinatal death [15]. Another study found that well-controlled PH, particularly in long-term responders to calcium channel blockers, were factors of better maternal outcomes [16]. In our series, diagnosis after the third trimester, and severe PH and/or right ventricular systolic dysfunction were determined to be maternal predictive factors of adverse fetal/neonatal events, Importantly, a history of prior cardiac events and right ventricular systolic dysfunction and/or baseline NYHA function class III or IV were the main determinants of adverse maternal cardiac events.

Due to the high mortality, pregnancy is strongly discouraged in patients with baseline NYHA functional class III or IV or severe PH [8, 9]. Termination in the first trimester is a safer option. Termination of pregnancy in its mid and late phases, with the concomitant volume and hormonal fluctuations, also poses a high risk to the mother. It may be reasonable to continue a pregnancy, however, after the risks of termination are balanced against the risks of continuation of the pregnancy.

It is noted that PH presence late in pregnancies could pose extreme difficulty in treatment. Patients with PH should be cared for by a systematic multidisciplinary team including cardiologists, obstetricians, maternal-fetal medicine, cardiac ultrasound specialists, cardiology service lines, cardiac surgery, cardiac anesthesiologists, and neonatal specialists to minimize maternal and fetal mortality [17, 18].

The timing and mode of delivery remain controversial. Scheduled cesarean delivery during combined spinal-epidural anesthesia has been reported to be a preferable choice [19]. For the 64 ongoing pregnancies in our series, 62 (96.9%) were delivered by caesarean section. Epidural anesthesia was the most commonly used technique in the study population.

Several limitations should be taken into account when interpreting the results of the study. First, the hemodynamic data were collected retrospectively. Hemodynamic data before pregnancy were often not available. Second, due to the invasiveness and high risk, right heart catheterization did not perform routinely on pregnant women in our institute. The estimation of pulmonary artery pressure was based on the Doppler pressure gradients across the tricuspid valve. Third, a referral bias may exist as a consequence that patients included in this study were treated at a tertiary care center. Finally, the small sample size in the present study could also affect the statistical analysis. A further prospective study with larger sample size and more sensitive diagnostic methods is needed in the future.

## 5. Conclusions

In this study, we found that pregnancy in women with PH was associated with significant cardiac and neonatal morbidity. Early diagnosis was essential for a better outcome. A pre-pregnancy baseline assessment could identify women at high risk for adverse outcomes. Pregnancy should therefore be strongly discouraged in patients with baseline NYHA functional class III or IV or severe PH and/or right ventricular systolic dysfunction. Cardiac interventions should be considered before conception for women with high risk for cardiac events.
